# The effect of gum Arabic supplementation on cathelicidin expression in monocyte derived macrophages in mice

**DOI:** 10.1186/s12906-022-03627-9

**Published:** 2022-06-01

**Authors:** Nagat Siednamohammeddeen, Rehab Badi, Tahane Mohammeddeen, Khalid Enan, AmalSaeed

**Affiliations:** 1 Department of Physiology, Faculty of Medicine, Red Sea University, Portsudan, Sudan; 2 Physiology Department, College of Medicine, King Khalid University, Abha, Saudi Arabia; 3 Department of Physiology, Faculty of Medicine, University of Khartoum, Khartoum, Sudan; 4 Department of Microbiology, Faculty of Medicine, Red Sea University, Portsudan, Sudan; 5 Department of Virology, Central Laboratory, Ministry of Higher Education and Scientific Reaserch, Khartoum, Sudan

**Keywords:** Cathelicidin, LL-37, Gum Arabic, Antimicrobial peptide, Butyrate

## Abstract

**Background:**

Antimicrobial peptides (AMPs) are important effectors of the innate defense system. Cathelicidins, (CRAMP in mouse/rat, LL-37 in human) is one of the two major classes of AMPs in humans. The upregulation of LL-37 synthesis is a novel non-antibiotic approach to prevent or treat infectious diseases. Butyrate was found to induce Cathelicidin expression. Gum Arabic (GA), an exudate from *Acacia senegal*tree, is known for its prebiotic effects. Fermentation of GA by colonic bacteria increases serum butyrate concentrations. This study was conducted to investigate if GA supplementation can increase Cathelicidin expression in macrophages.

**Methods:**

The study was an in-vivo experiment in mice. Thirty mice were randomly divided into three groups, ten mice per group. The two intervention groups received GA dissolved in drinking water in two different concentrations (15% w/v and 30% w/v) for 28 days. The third group served as a control. Blood was collected on Day 29 to isolate peripheral blood mononuclear cells (PBMC) which were cultured to obtain monocyte derived macrophages (MDMs). The transcription level of CRAMP was determined in MDMsby qPCR.

**Results:**

We detected a significant increase (*p* = 0.023) in CRAMP expression in MDMs following 28 days of 15% GA supplementation, compared to the control group, but there was no significant change in the group on 30% GA supplementation (*p* = 0.055).

**Conclusion:**

GAsupplementation can induce Cathelicidin expression in MDMs and the effect is dose dependent.

**Supplementary Information:**

The online version contains supplementary material available at 10.1186/s12906-022-03627-9.

## Introduction

Antimicrobial peptide (AMPs), also known as host defense peptides (HDPs), act as natural broad-spectrum antibiotics and play essential roles in regulating host defense and immunity [1, 2]. More than hundred human AMPs have been identified from various tissues and epithelial surfaces [3]. Nevertheless most human AMPs belong to two major families: the Defensins and the Cathelicidins [4]. Members of theCathelicidins family of AMPs share a conserved N-terminal Cathelin domain [5]. Cathelicidins were first identified in bone marrow myeloid cells, therefore they were referred to as”myeloid antimicrobial peptides” (MAP) [6, 7]. In humans, the only Cathelicidin is the 37 amino acid peptide LL-37. This peptide is expressedin many immune cells such as neutrophils, monocytes/macrophages and lymphocytes [5]. LL-37 is also expressed in epithelial cells of the intestine, airways, skin, and the urogenital tract [8–10]. Cathelicidin is stored inside cellular granules in an inactive precursor form and is activated upon secretion by proteolitic cleavage of the conserved cathelin domain from the active C-terminal region [6].

LL-37 has broad-spectrum antimicrobial activity against Gram-positive and Gram-negative bacteria, as well as against fungi and enveloped viruses [11]. The anti-infective properties of LL-37 depend on its direct microbicidal and immunomodulatory activities. The direct microbicidal activity of LL-37 is predominantly mediated by disrupting the integrity of microbial membranes, due to its inherent cationic and amphipathic nature [12]. Additionally, a multi-hit mechanism in which the peptide translocates through the bacterial inner membrane to interacts with several intracellular targets appears to enhance direct microbicidal activity [13]. For example in *Escherechia coli* intracellular LL-37 induced the production of reactive oxygen species (ROS), increased the permeability of the membrane and affected the transcription of genes related to energy production and carbohydrates metabolism with the net result of inhibiting bacterial growth [14].

Despite its direct microbicidal action, several line of evidence have shown that the anti-infective activity of LL-37 in vivo is mainly dependent on its ability to modulate the immune response [15]. LL-37 boosts immune-activating functions such as chemokine production, leukocyte recruitment, immune cell activation, T-cell polarization and angiogenesis [16, 17]. Also, LL-37 induces autophagy-mediated killing which provides protection against infection with intracellular organisms such as *Mycobacterium tuberculosis* [18].

The non-microbicidal, immunomodulatory actions of Cathelicidins have attracted increasing attention and have been associated with several autoimmune diseases; such as psoriasis [19], systemic lupus erythematous [20], arthritis [21], atherosclerosis [22, 23], and type 1 diabetes [24]. Interestingly a study showed that the gut microbiota via short-chain fatty acids promoted the production of mCRAMP (mouse cathelicidin-related antimicrobial peptide), the only cathelicidin in rats and mice, by pancreatic endocrine cells, which provided protection against autoimmune diabetes by inducing regulatory immune cells in the pancreas [24].

Induction of endogenous CRAMP/LL-37 expression has emerged as a novel approach in combating infectious diseases through alternative therapy.

Short-chain fatty acids, butyrate in particular, were found to be strong inducers of LL-37 gene expression [25]. Sodium phenyl-butyrate (PBA) is a registered drug used for the treatment of urea cycle disease [26]. Earlier studies have shown that treatment with PBA enhanced cathelicidin expression in a colonic epithelia cell line and in colonic epithelium in a rabbit model of shigellosis, which resulted in rapid clinical recovery and a concomitant decline in bacterial load in stool [27, 28]. Similarly, studies in rabbit model of shigellosis have further shown that PBA treatment overcome Shigella mediated down regulation of Cathelicidin expression in in a bronchial epithelial cells [29, 30].

An in vitro study showed that PBA induced cellular production of LL-37and inhibited the growth of *Mycobacterium tuberculosis* (Mtb) in human macrophages [31]. Furthermore, an in vivo study showed that oral intake of PBA up regulates LL-37 in human macrophages and lymphocytes, and induced intracellular killing of Mtb [32]. In a randomized controlled trial oral PBA together with vitD,which is also known to induce LL-37 expression, demonstrated beneficial effects toward clinical recovery in tuberculosis patients [33]. .

Gum Arabic (GA) is derived from the exudates of *Acacia senegal* or *Acacia seyal* trees. It consists of a mixture of polysaccharides (major component) plus oligosaccharides and glycoproteins [34, 35]. Oral intake of GA has been shown to provide several health benefits [36]. GA significantly increases *Bifidobacteria, Lactobacteria, and Bacteriodes* in the gut and is considered to have prebiotic actions [37]. GA is claimed to have anti-cancer [38], anti-malarial [39] immune-modulatory [38, 39] and antioxidant effects [37, 40]. GA treatment has been shown to favorably influence clinical and laboratory results in rats with adenine-induced chronic renal failure (CRF) and in humans diagnosed with renal failure [40–42]. It also increased Erythropoietin level in two separate studies and ameliorated anemia induced by adenine administration [43, 44]. Very recently GA was found to have a novel effect on fetal hemoglobin production [45]. Also, Gum Arabic ingestion resulted in decreased inflammatory markers and disease severity score among Rheumatoid Arthritis Patients [46].

Regarding Cathelicidin expression, several studies showed that GA ingestion increases serum short chain fatty acid concentration, mainly butyrate and propionate [47, 48].. Many of the health beneficial effects of GA mentioned above was related to increased serum butyrate concentration following regular GA intake [33]. Increasing Cathelicidin expression is beneficial in treatment of diseases such as shigellosis and tuberculosis in which Cathelicidin expression is down regulated as discussed earlier [28, 33]. Also Cathelicidin through its immunomodulatory functions was involved in several autoimmune diseases and cardiovascular diseases [22–24]. And since butyrate is a potent inducer of Cathelicidin expression, we were interested in investigating the effect of regular GA intake on Cathelicidin expression by immune cells.

## Material and method

### Gum Arabic

Gum Arabic powder used in the study was a 100% natural powder produced mechanically from the extract of *Acacia Senegal* tree with a particle size less than 210 μm (Dar Savanna Ltd., Khartoum, Sudan). Its quality was consistent to the requirements of Food and Agriculture Organization of the United Nations (FAO) and British pharmacopoeia (BP).

### Animal handling and the experimental groups

The study included 30 wild-type male mice aged 4 months, with an average weight of 25 g. Mice were obtained from the National Center For Research (Khartoum, Sudan)and were housed in separate cages. Mice had free access to food and water. After 5 days adaptive period, mice were randomly and equally divided into three groups as follow: The control group; 15% GA group: mice in this group received 30 g/d GA in 200 ml drinking water in a concentration of 15%w/v; and 30% GA group: mice in this group received 60 g/d GA in 200 ml drinking water in a concentration of 30% w/v. GA was provided to the two intervention groups. The lower dose of GA was selected based on previous experiments with GA [42, 44]. The higher dose was used to investigate if the effect of GA was dose related. GA supplementation continued for 28 days in which fresh GA solutions were prepared daily. Mice were weighed weekly. Animals were checked at least daily, even on weekends. At the end of the 28 days period, mice were euthanized via CO_2_ inhalation and blood samples were collected under aseptic condition.

### Blood collection and cell culture

Blood samples were collected on day 29.Mice were euthanized and blood collected by cardiac puncture and placed into EDTA tubes. Samples were centrifuged for 10 minutes at 2000 g and the plasma was stored at − 20 C°for further analysis. The buffy coat was transferred into a single tube that can contain at least twice its volume. Then it was diluted and mixed gently with equal amount of PBS.

### PBMC isolation and culture

Peripheral blood mononuclear cells (PBMC) were isolated from the diluted buffy coat using Ficoll–Paque density gradient (3 ml of Ficoll) was added to the bottom of a15 ml conical tube without touching the side of the tube. Gently the Ficoll was overlayed with the diluted buffy coat. The diluted sample was allowed to flow down the side of the tube and pool on top of the density gradient media surface without breaking the surface plane and then the tube was centrifuged at 400 g for 20 min at 18 °C. New sterile conical tubes were labeled, with the same number and same volumes as for the density gradient cell separation, which will contain PBMCs. The plasma layer was removed as much as possible with pipette. The mononuclear cells were collected from the plasma/Ficoll interface with a disposable transfer pipette and transferred into a new sterile conical tube. PBS (3 ml) was added to each tube and the tubes were centrifuged at 400 g for 10 minutes. The supernatant was removed carefully (the pellet is loose because of red blood cells) by tilting the tube and pipetting off the supernatant without touching the pellet. The cell pellet was suspended by gentle pipetting in a 1 mL PBS and the tube was then filled with PBS and centrifuged at 400 g for 10 minutes. The supernatant was discarded and cell concentration and viability were determined by using the Cellometer 2000. PBMCs were washed by using PBS and re-suspended in culture medium containing 10% autologous plasma in RPMI-1640, 1% L-glutamine, 1% sodium pyruvate, 0.5% amphotericin B and 1% penicillin-streptomycin. Cells were plated in two parallel 4-well tissue culture plates (NUNC, Roskilde, Denmark) and incubated for 5 days in 5% CO_2_ at 37 °C. Thereafter, the supernatant containing the non-adherent cells was removed. The remaining adherent cells in the culture plate monocyte derived macrophage (MDM) were harvested using a cell scraper and treated with Saponin 0.1% [32].

### Quantitative real time RT-PCR amplification of CRAMP

RNA was extracted from MDM samples as follow: Cells were homogenized in TRI Reagent (easy- BlueTM Total RNA extraction kit) then it was extracted with chloroform in phase lock tubes (5 Prime) following the manufactures protocol and stored at − 80 °C until processed. RNA integrity and concentration were determined on a NanoDrop 2000 (Thermo Scientific). The b-actin primers used to amplify beta actin were, BACTIN-1 (GGT-CGTCGACAACGGCTC) and BACTIN-2 (TGCCATGTTCAATG-GGGTAC). CRAMP-F1 (5′-AGGAGATCTTGGGAACCATGCAGTT-3′) and CRAMP-R1 (5′-GCAGATCTACTGCTCCGGCTGAGGTA-3′) were the primers used to amplify CRAMP [49]. The reverse transcriptase reactions were run with reverse transcription reaction template and oligos using SYBR Green PCR Master Mix (iNtRON Biotechnology). All measurements were performed in triplicate with Rotor- Gene Q Series software 2.3.1 sequence detection system. The thermal profile consisted of 42 °C for 20 min., 95°Cfor 10 min., followed by 45 cycles of 95 °C for 10 s and 60 °C for 60 seconds. Results were analyzed by the comparative cycle threshold (Ct) Method, where Ct is the number of cycles required to reach an arbitrary threshold [49].

### Statistics

Data was analyzed using statistical package for social sciences (SPSS ver. 22). Kruskal-Wallis ANOVA on Ranks test was used to compare groups..The test was considered significant, when P. value was less than 0.05. Results were expressed as mean and standard deviation.**.**

## Results

### Effects of GA supplementation on cathelicidin expression in mice monocyte derived macrophages

**Fig. 1:**
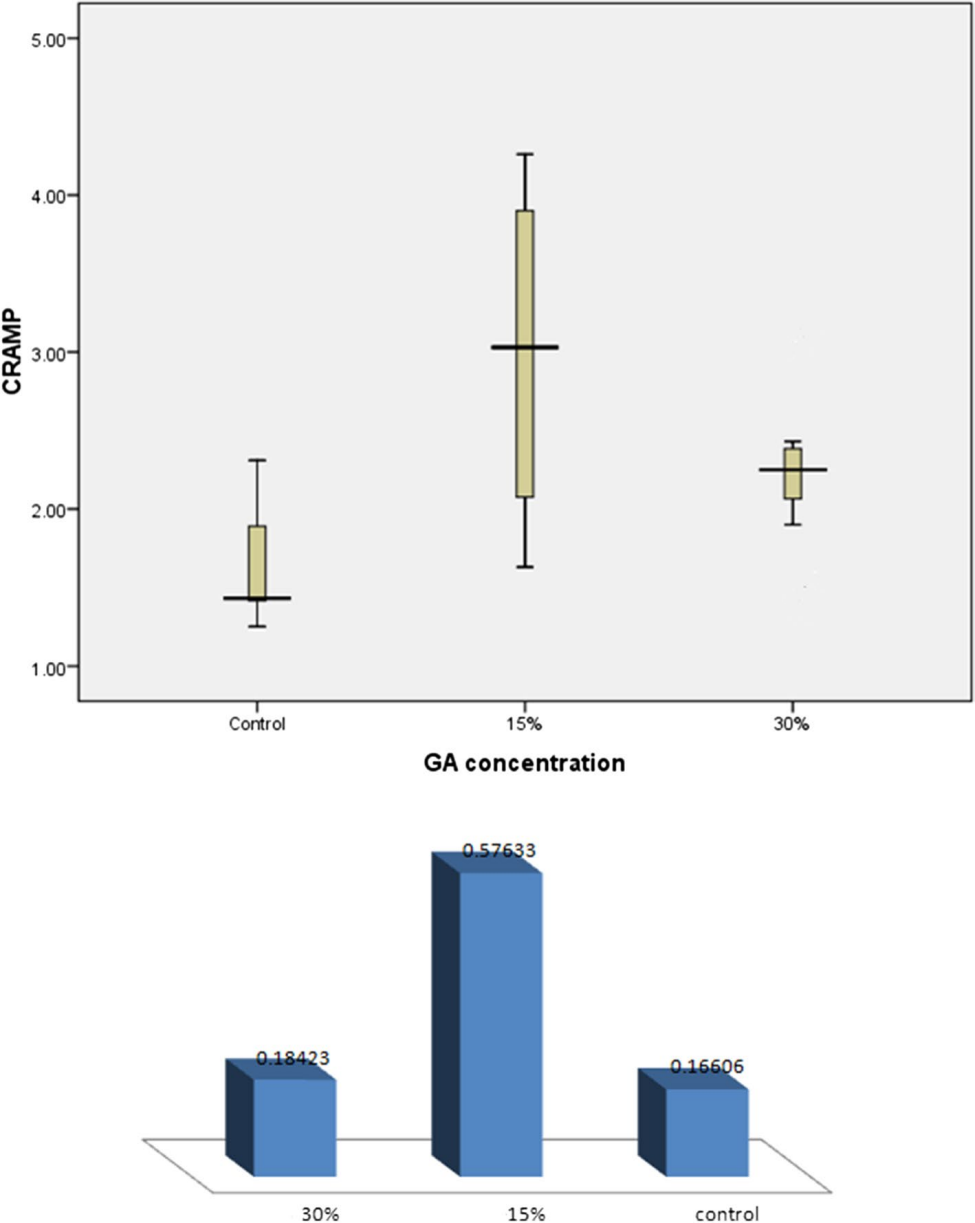
Effect of Gum Arabic supplementation on CRAMP expression. Note: Group-I: control group; Group-II: 15% GA supplementation; Group-III: 30% GA supplementation. In the 15% GA and 30% GA groups, mice received GA dissolved in their drinking water in a concentration of 15% w/v or 30% w/v respectively for 28 days. Data expressed as Mean Square. Differences are significant when *p* < 0.05. There is significant difference in the mean of CRAMP before and after GA supplementation in 15% GA group (*p* = 0.023)

Comparison between the three groups regarding the levels of CRAMP constructs revealed significant difference (*p* = 0.028) (Fig. 1). The group that was supplemented with 15% GA showed significantly higher levels of CRAMP transcripts compared to the control group (*p* = 0.023). Although the levels of CRAMP transcripts increased in the group that received 30% GA they were not statistically different from the control group (*p* = 0.055).

## Discussion

Several cell types including macrophage are known to express Cathelicidin [3, 7]. The aim of the study was to evaluate the effect of GA supplementation on Cathelicidin expression in mice MDMs. We administrated GA to two groups of healthy mice in two different concentrations: 15 and 30% w/v for 28 days. The expression of CRAMP in MDM cellswas assessed using Quantitative real time PCR. Our results showed that there was a significant increase in RNA expression of Cathelicidin among the groups supplemented with GA in a concentration of 15% (*P* = 0.023) in comparison with the control group. These results were expected since GA has been shown to increase circulating butyrate concentration. Matsumoto’set. al, showed that ingestion of 25 g of GA daily doubled serum butyrate level [47]. Butyrate is known to increase cathelicidin production in immune cells [31]. It has been shown that the downregulation of the rabbit cathelicidin (CAP-18) in the colonic and lung epithelium can be opposed by oral treatment with sodium butyrate or its analogue, PBA, in experimental model of shigellosis [28, 30]. Both butyrate and PBA induced LL-37 expression in human lung epithelial and colonic epithelial lines [29].

Studies have shown that butyrate affects LL-37 expression mainly at the transcriptional level and that LL-37 mRNA expression was directly correlated with LL-37 protein synthesis and antimicrobial activity both in vitro and in vivo [50]. Hyperacetylation of core histones is considered to be the major mechanism of butyrate-induced LL-37 expression [51]. Nevertheless, it has been shown that MAP kinase signaling pathway is also involved in butyrate- induced CAMP gene expression [51].

Cathelicidin RNA expression in MDMs obtained from mice receiving high concentrations of GA (30% w/v.) was higher when compared to the control group, but the difference was not statistically significant Fig. 1). This indicates that butyrate induced CRAMP expression was dose dependent, and the stimulatory effect of butyrate is lost in higher butyrate concentrations [52]. As reported by Soliman et al. butyrate up-regulated leptin expression within physiological levels (1 mM) but the high doses inhibited gene expression of Leptin. It is uncertain why high doses of butyrate exert this inhibitory effect.

In conclusion, the study demonstrated a novel effect of GA, as an inducer for cathelicidin expression. Being able to induce the expression of cathelicidin, regular consumption of GA can therefore enhance the innate immunity against bacterial, viral, and fungal infections. Hence the study asserts that GA is a promising candidate as an adjuvant treatment in infectious diseases. Effect of GA supplementation in Cathelicidine expression in humans should be investigated.

## Supplementary Information

**Additional file 1.**

**Additional file 2.**

## Data Availability

The datasets used and /or analysed during the current study available from the corresponding author on reasonable request.
