# Supplementary material for: The effect of gum Arabic supplementation on cathelicidin expression in monocyte derived macrophages in mice
Source: BMC Complement Med Ther. 2022 Jun 1;22:149. doi: 10.1186/s12906-022-03627-9 (PMC9158159; doi:10.1186/s12906-022-03627-9)
Supplement: Supplementary file 1 — Additional file 1. [file 12906_2022_3627_MOESM1_ESM.docx]

Normality test

| **Descriptives** | | | | | |
| --- | --- | --- | --- | --- | --- |
|  | type | | | Statistic | Std. Error |
| CRAMP | Control | Mean | | 1.6571 | .16606 |
|  |  | 95% Confidence Interval for Mean | Lower Bound | 1.2508 |  |
|  |  |  | Upper Bound | 2.0635 |  |
|  |  | 5% Trimmed Mean | | 1.6435 |  |
|  |  | Median | | 1.4300 |  |
|  |  | Variance | | .193 |  |
|  |  | Std. Deviation | | .43934 |  |
|  |  | Minimum | | 1.25 |  |
|  |  | Maximum | | 2.31 |  |
|  |  | Range | | 1.06 |  |
|  |  | Interquartile Range | | .86 |  |
|  |  | Skewness | | 1.094 | .794 |
|  |  | Kurtosis | | -.888- | 1.587 |
|  | 15% | Mean | | 2.9875 | .57633 |
|  |  | 95% Confidence Interval for Mean | Lower Bound | 1.1534 |  |
|  |  |  | Upper Bound | 4.8216 |  |
|  |  | 5% Trimmed Mean | | 2.9922 |  |
|  |  | Median | | 3.0300 |  |
|  |  | Variance | | 1.329 |  |
|  |  | Std. Deviation | | 1.15266 |  |
|  |  | Minimum | | 1.63 |  |
|  |  | Maximum | | 4.26 |  |
|  |  | Range | | 2.63 |  |
|  |  | Interquartile Range | | 2.23 |  |
|  |  | Skewness | | -.163- | 1.014 |
|  |  | Kurtosis | | -1.870- | 2.619 |
|  | 30% | Mean | | 2.2286 | .18423 |
|  |  | 95% Confidence Interval for Mean | Lower Bound | 1.7778 |  |
|  |  |  | Upper Bound | 2.6794 |  |
|  |  | 5% Trimmed Mean | | 2.2290 |  |
|  |  | Median | | 2.2500 |  |
|  |  | Variance | | .238 |  |
|  |  | Std. Deviation | | .48742 |  |
|  |  | Minimum | | 1.43 |  |
|  |  | Maximum | | 3.02 |  |
|  |  | Range | | 1.59 |  |
|  |  | Interquartile Range | | .53 |  |
|  |  | Skewness | | -.079- | .794 |
|  |  | Kurtosis | | 1.317 | 1.587 |

| **Tests of Normality** | | | | | | | |
| --- | --- | --- | --- | --- | --- | --- | --- |
|  | type | Kolmogorov-Smirnov^a^ | | | Shapiro-Wilk | | |
|  |  | Statistic | df | Sig. | Statistic | Df | Sig. |
| CRAMP | Control | .345 | 7 | .012 | .753 | 7 | .014 |
|  | 15% | .184 | 4 | . | .981 | 4 | .906 |
|  | 30% | .215 | 7 | .200^*^ | .955 | 7 | .776 |
| a. Lilliefors Significance Correction | | | |  |  |  |  |
| *. This is a lower bound of the true significance. | | | | |  |  |  |

رسمة الصندوق


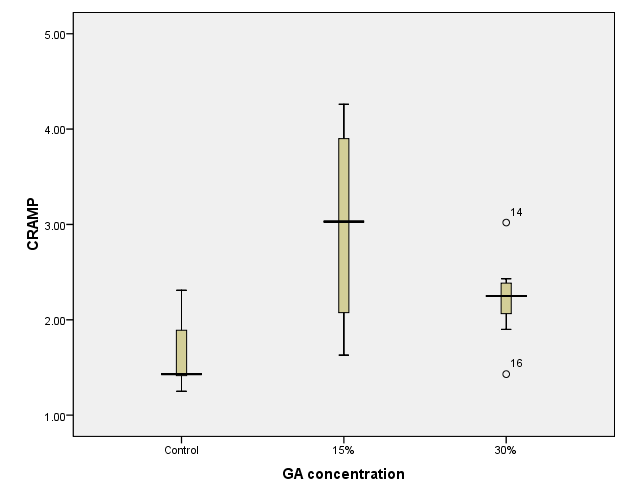


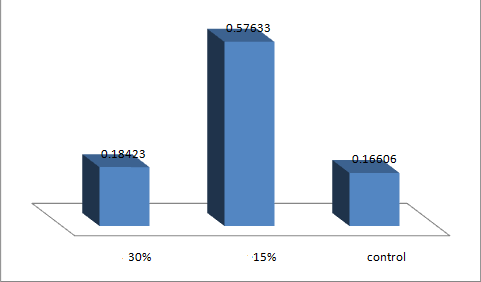


المقارنة للمجموعات الثلاث:

| **Ranks** | | | |
| --- | --- | --- | --- |
|  | type | N | Mean Rank |
| CRAMP | Control | 7 | 5.64 |
|  | 15% | 4 | 14.25 |
|  | 30% | 7 | 10.64 |
|  | Total | 18 |  |

| **Test Statistics^a,b^** | |
| --- | --- |
|  | CRAMP |
| Chi-Square | 7.149 |
| df | 2 |
| Asymp. Sig. | .028 |
| a. Kruskal Wallis Test | |
| b. Grouping Variable: type | |

اختبار مان وتني:

| **Ranks** | | | | |
| --- | --- | --- | --- | --- |
|  | type | N | Mean Rank | Sum of Ranks |
| CRAMP | Control | 7 | 4.29 | 30.00 |
|  | 15% | 4 | 9.00 | 36.00 |
|  | Total | 11 |  |  |

| **Test Statistics^b^** | |
| --- | --- |
|  | CRAMP |
| Mann-Whitney U | 2.000 |
| Wilcoxon W | 30.000 |
| Z | -2.268- |
| Asymp. Sig. (2-tailed) | .023 |
| Exact Sig. [2*(1-tailed Sig.)] | .024^a^ |
| a. Not corrected for ties. | |
| b. Grouping Variable: type | |

.023 <.05

توجد فروق بين 15% & control


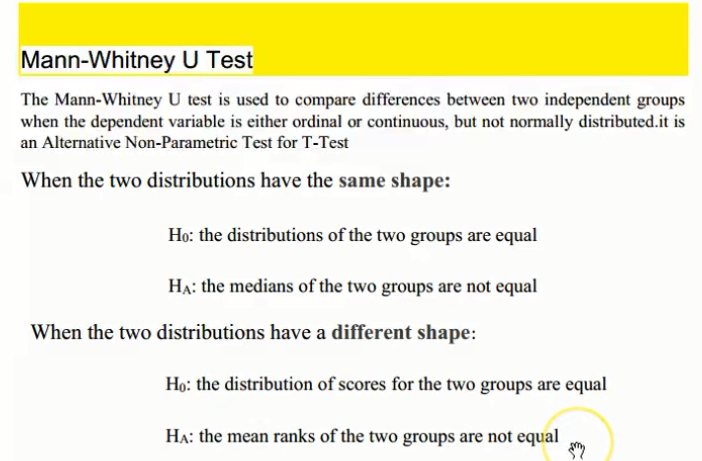


| **Ranks** | | | | |
| --- | --- | --- | --- | --- |
|  | type | N | Mean Rank | Sum of Ranks |
| CRAMP | Control | 7 | 5.36 | 37.50 |
|  | 30% | 7 | 9.64 | 67.50 |
|  | Total | 14 |  |  |

| **Test Statistics^b^** | |
| --- | --- |
|  | CRAMP |
| Mann-Whitney U | 9.500 |
| Wilcoxon W | 37.500 |
| Z | -1.919- |
| Asymp. Sig. (2-tailed) | .055 |
| Exact Sig. [2*(1-tailed Sig.)] | .053^a^ |
| a. Not corrected for ties. | |
| b. Grouping Variable: type | |

| **Ranks** | | | | |
| --- | --- | --- | --- | --- |
|  | type | N | Mean Rank | Sum of Ranks |
| CRAMP | 15% | 4 | 7.75 | 31.00 |
|  | 30% | 7 | 5.00 | 35.00 |
|  | Total | 11 |  |  |

| **Test Statistics^b^** | |
| --- | --- |
|  | CRAMP |
| Mann-Whitney U | 7.000 |
| Wilcoxon W | 35.000 |
| Z | -1.323- |
| Asymp. Sig. (2-tailed) | .186 |
| Exact Sig. [2*(1-tailed Sig.)] | .230^a^ |
| a. Not corrected for ties. | |
| b. Grouping Variable: type | |
