# Supplementary material for: The effect of gum Arabic supplementation on cathelicidin expression in monocyte derived macrophages in mice
Source: BMC Complement Med Ther. 2022 Jun 1;22:149. doi: 10.1186/s12906-022-03627-9 (PMC9158159; doi:10.1186/s12906-022-03627-9)
Supplement: Supplementary file 2 — Additional file 2. [file 12906_2022_3627_MOESM2_ESM.doc]

| 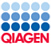 | [www.qiagen.com](http://www.qiagen.com/) |
| --- | --- |

# Quantitation Report

#### Experiment Information

| Run Name | CRAMP 2019-02-06(1) |
| --- | --- |
| Run Start | 06/02/2019 1:42:52 PM |
| Run Finish | 06/02/2019 3:51:00 PM |
| Operator | Janine |
| Notes |  |
| Run On Software Version | Rotor-Gene Q Software 2.3.1.49 |
| Run Signature | The Run Signature is valid. |
| Gain Green | 10. |
| Gain Yellow | 10. |
| Gain Orange | 10. |
| Machine Serial No. | 1014309 |

#### Quantitation Information

| Threshold | 0.020 |
| --- | --- |
| Left Threshold | 1.000 |
| Standard Curve Imported | Yes |
| Standard Curve (1) | conc= 10^(0.122*CT + -2.694) |
| Standard Curve (2) | CT = 8.196*log(conc) + 22.082 |
| Reaction efficiency (*) | (* = 10^(-1/m) - 1) -0.24493 |
| Start normalising from cycle | 1 |
| Noise Slope Correction | Yes |
| No Template Control Threshold | % 0 |
| Reaction Efficiency Threshold | Disabled |
| Normalisation Method | Dynamic Tube Normalisation |
| Digital Filter | Light |
| DynamicTubeOptimisation | 15 20 |
| Sample Page | Page 1 |
| Imported Analysis Settings |  |

#### Profile

| Cycle | Cycle Point |
| --- | --- |
| Hold 1 | Hold @ 42°C, 20min 0s |
| Hold 2 | Hold @ 95°C, 10min 0s |
| Cycling (45 repeats) | Step 1: Hold @ 95°C, 10s |
|  | Step 2: Hold @ 60°C, 60s, acquiring to Cycling A([Green][1][1],[Orange][3][3],[Yellow][2][2]) |

#### Raw Data For Cycling A.Green


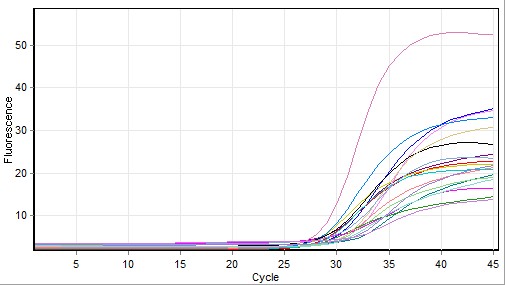


#### Quantitation data for Cycling A.Green


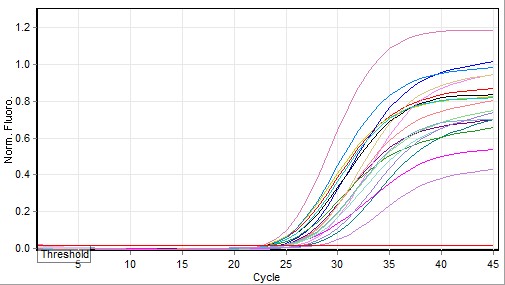


#### Standard Curve


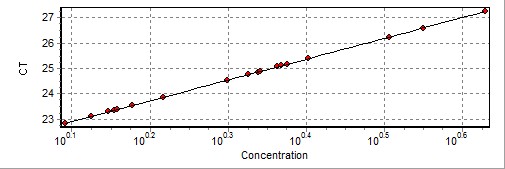


| No. | Color | Name | Type | Ct | Ct Comment | Given Conc (copies/ul) | Calc Conc (copies/ul) |
| --- | --- | --- | --- | --- | --- | --- | --- |
| 1 | 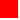 | 2 | Unknown | 23.29 |  |  | 1.42024252243683 |
| 2 | 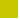 | 3 | Unknown | 23.11 |  |  | 1.41111846478515 |
| 3 | 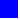 | 4 | Unknown | 24.53 |  |  | 1.90058172623843 |
| 4 | 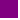 | 5 | Unknown | 24.86 |  |  | 2.27147589979768 |
| 5 | 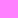 | 6 | Unknown | 26.23 |  |  | 3.0294695651489 |
| 6 | 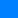 | 7 | Unknown | 23.38 |  |  | 1.4302983020078 |
| 7 | 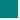 | 8 | Unknown | 27.24 |  |  | 4.26382475940548 |
| 8 | 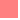 | 9 | Unknown | 25.06 |  |  | 2.31121720278207 |
| 9 | 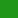 | 10 | Unknown | 23.86 |  |  | 1.6334841336978 |
| 10 | 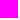 | 11 | Unknown | 24.88 |  |  | 2.2581078042985 |
| 11 | 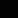 | 12 | Unknown | 23.35 |  |  | 1.43757161432293 |
| 12 | 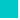 | 13 | Unknown | 23.54 |  |  | 1.51045712163395 |
| 13 | 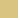 | 14 | Unknown | 25.10 |  |  | 2.43707116266248 |
| 14 | 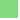 | 15 | Unknown | 25.17 |  |  | 2.345654322160157 |
| 15 | 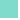 | 16 | Unknown | 25.38 |  |  | 2.52543933487776 |
| 16 | 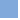 | 17 | Unknown | 24.76 |  |  | 2.23062043409648 |
| 17 | 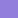 | 18 | Unknown | 26.59 |  |  | 3.54761642236441 |
| 18 | 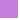 | 19 | Unknown |  | NEG (Multi Ct) |  |  |
| 19 | 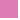 | -ve | NTC | 22.83 |  |  | 1.25219229574997 |

**Legend:**
NEG (NTC) - Sample cancelled due to NTC Threshold.
NEG (R. Eff) - Sample cancelled as efficiency less than reaction efficiency threshold.

| This report was generated by Rotor-Gene Q Series Software 2.3.1 (Build 49) Copyright 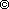2013 QIAGEN GmbH. All Rights Reserved. |
| --- |
